# Pasture Types and *Echinococcus multilocularis*, Tibetan Communities

**DOI:** 10.3201/eid1206.041229

**Published:** 2006-06

**Authors:** Qian Wang, Dominique A. Vuitton, Yongfu Xiao, Christine M. Budke, Maiza Campos-Ponce, Peter M. Schantz, Francis Raoul, Wen Yang, Philip S. Craig, Patrick Giraudoux

**Affiliations:** * Sichuan Provincial Center for Disease Control and Prevention, Chengdu, Sichuan, People's Republic of China;; † University of Franche-Comte, Besançon, France;; ‡ Texas A & M University, College Station, Texas, USA;; § Free University, Amsterdam, the Netherlands;; ¶ Centers for Disease Control and Prevention, Atlanta, Georgia, USA;; # University of Salford, Salford, United Kingdom

**Keywords:** Echinococcus multilocularis, pasture type, pastoralist communities, mammals, alveolar echinococcosis, dispatch

## Abstract

Our study showed that open pastures had more small mammal burrows than fenced pastures in Tibetan pastoralist communities in 2003. This characteristic was linked to a higher prevalence of *Echinococcus multilocularis* in dogs and indicates that pasture type may affect *E. multilocularis* transmission.

Human alveolar echinococcosis (AE) is an infection caused by *Echinococcus multilocularis*, a highly pathogenic cestode. Foxes are frequently definitive hosts (adult stage), and small mammals are intermediate hosts (larval stage or metacestode). Human AE, albeit restricted to localized endemic areas, is a public health concern in central Europe (*1*). In western China (*2**,**3*), dogs are definitive hosts of AE and have transmitted infection to humans more often than was realized historically (*4*). Recent mass ultrasound screenings in Tibetan pastoral communities of Sichuan Province, People's Republic of China, documented an average AE prevalence of 2% (*5*) and a maximum prevalence of 14.3% (*6*) in humans.

Since the 1980s, partial fencing of pastures around Tibetan pastoral winter settlements has become common because of changes in land property regulations (*7*). In a previous study, we showed that partial fencing promoted AE transmission in these communities (*8*). This increased disease prevalence is likely due to the greater population of small mammal intermediate hosts of the parasite on the Tibetan plateau and leads to more infection in community dogs (*8*). When yak, sheep, and horse grazing lowers the height of vegetation, *Ochotona curzoniae*, a very susceptible host for *E. multilocularis*, may be found at greater densities than on natural meadows (*9*). This study was designed to investigate the effect of partial fencing on the general abundance of small mammals in the Tibetan pastoralist winter settlements and its potential consequences for contamination pressure. The study was approved by the ethical committees of Sichuan Institute of Parasitic Diseases and all collaborating investigators.

## The Study

Based on documented high prevalence of AE and observed fencing practices in the area (*8*), Qiwu, Yiniu, and Xiazha townships in Shiqu County of Ganzi Tibetan Autonomous Prefecture, located at a mean elevation of 4,200 m in northwest Sichuan Province, were selected as study sites to carry out investigations in spring and autumn 2003. For the 3 townships, the populations were 2,238, 2,515, and 2,471 and the areas 1,046 km^2^, 955 km^2^, and 834 km^2^, respectively. Thirty kilometers of transect over 30 settlements in the 3 townships (18 villages), which were randomly selected according to landscape patterns, i.e., valley, valley entrance, piedmont, and flat land, were surveyed. Small mammal populations were monitored by using index methods. These methods are based on detecting surface indicators of small mammals, i.e., holes and feces, and are used to link small mammals and their habitats on large areas (*10**–**12*). Sampling was performed by 2 investigators walking along a 1-km transect drawn across each settlement, according to a standardized protocol. Along each transect, 50 areas were sampled for small mammal burrows; each area was 200 m^2^. Areas of fenced pastures were measured in 22 settlements by using a global positioning system (GPS) (GPS 12, Garmin International Inc., Olathe, KS, USA). In 15 settlements in which dog feces samplings were conducted in Yiniu and Xiazha townships, feces specimens were collected from dogs after purging with arecoline, according to the recommendations of World Animal Health Organization/World Health Organization (*13*), and droppings were collected from the ground when accessible (*4*). Helminths found in the feces were removed, counted, and placed in 10% formal saline or 85% ethanol. Copro-polymerase chain reaction testing was conducted by using species-specific primers for *E. multilocularis* DNA amplification according to Dinkel et al. (*14*) as modified by van der Giessen et al. (*15*).

## Conclusions

The distribution of small mammal burrows was highly skewed. Kolmogorov-Smirnov test indicated that the data did not fit a normal distribution (p<0.001 in both cases) either inside or outside fenced pastures. Normality was not obtained after Box-Cox transformations. Thus, the burrow density of small mammals was compared between open and fenced pastures by using nonparametric tests that considered landscape factor. Spearman correlation tests were used to quantify the relationship between the burrow density of small mammals on open pastures and the surface of fenced pastures in settlements in which the fenced areas were all measured, controlling for the landscape factor. A multiple logistic regression model was used to relate median burrow density of small mammals to dog infection in the settlements. The dependent variable was a presence/absence vector (0/1) (dog was negative or positive for *E. multilocularis*). Independent variables included dog's age and sex, droppings collected versus purged fecal samples, and median density of small mammal burrows. All these analyses used SPSS release 10 (SPSS, Chicago, IL, USA).

Landscape type influenced the abundance of small mammal burrows (p<0.001). Post hoc Tukey multiple comparison test on ranks confirmed that the densities of small mammal burrows were different among different landscape types (p<0.05), except for the comparison between flatland and piedmont. The burrow densities of small mammals on open pastures were significantly higher than those on fenced pastures in 3 of 4 landscapes (Table 1).

**Table 1:** Comparisons of open and fenced pastures small mammal burrow densities, stratified by landscapes*

Landscape	Pasture type	No. observations	Mean rank of densities	Sum rank of densities	Mann-Whitney U	Z	Asymptomatic p (2-tailed)
Valley	Open	616	439.07	270,464.50	63,715.500	–2.784	0.005
	Fenced	234	389.79	91,210.50			
Flat land	Open	175	109.38	19,141.00	634.000	–5.819	<0.001
	Fenced	25	38.36	959.00			
Piedmont	Open	155	96.91	15,020.50	2,930.500	–1.643	0.100
	Fenced	45	112.88	5,079.50			
Valley entrance	Open	180	103.83	18,690.00	1,200.000	–2.833	0.005
	Fenced	20	70.50	1,410.00			

*Density, no. burrows per 200 m^2^ of pasture.

The Spearman correlations showed larger fenced areas associated with higher density of small mammal burrows in the open pastures in all landscape types (Table 2). The relationships between the area of fenced pastures and the burrow density of small mammals inside the fenced pastures in the 4 landscapes were not significant (valley [*r_s_* = –0.08, p = 0.32], flatland [*r_s_* = –0.46, p = 0.02], piedmont [statistics not applicable because of 3 observations only], and valley entrance [*r_s_* = 0.08, p = 0.736]), except for flatland.

**Table 2:** Relationship between surface of fenced pastures and densities of small mammal burrows in open pastures*

Characteristic	Valley	Flat land	Piedmont	Valley entrance
No. observations	490	126	147	130
Correlation coefficient	0.382	0.312	0.471	0.296
p (2-tailed)	<0.001	<0.001	<0.001	0.001

*Spearman correlations.

Feces samples, of which 159 (63.1%) were purged, were obtained from 252 dogs (mean age 4.4 years; 183 males). *E. multilocularis* infection rate was 16.7% (42/252); the infection rate was 18.2% (29/159) for purged samples and 14.0% (13/93) for sampled droppings. Multiple logistic regressions showed that the median density of small mammal burrows in the open pastures was significantly positively related to dog infection (p = 0.003, odds ratio 1.05, 95% confidence interval 1.02–1.08). No correlation to dog age (p = 0.52), sex (p = 0.78), or sample collection method was seen (p = 0.380).

The higher median burrow density of small mammals was linked to a higher prevalence of *E. multilocularis* in dogs in these Tibetan pastoralist communities. Thus, partial fencing increases populations of potentially susceptible small mammal species in open pastures and consequently higher contamination pressure by dogs.

In a previous study, we showed that partial fencing around Tibetan settlements in winter pasture was significantly and independently associated with the risk for human AE in surveyed villages (*8*). We assumed that the underlying reason might be overgrazing, exacerbated by reducing communal pastures near the settlements because of introduction of partial fencing around group tenure pastures acquired by Tibetan pastoralist families. Overgrazing may have promoted population outbreaks of small mammal intermediate hosts of the parasite and increased the density of the small mammal intermediate host, especially *O. curzoniae*. This increase consequently favored higher dog *E. multilocularis* infection and, thus, transmission to humans (*8*). This study supports this hypothesis.
